# Unusual erythematous plaque with white scales, a case of acquired syphilis in a child and literature review

**DOI:** 10.1186/s12879-021-06114-7

**Published:** 2021-06-05

**Authors:** Wen-Jia Yang, Hong-Hao Hu, Yang Yang, Jiu-Hong Li, Hao Guo

**Affiliations:** Department of Dermatology, The First Hospital of China Medical University, 155N. Nanjing Street, Shenyang, 110001 P. R. China

**Keywords:** Acquired syphilis, Preschool children, Nonsexual close contact

## Abstract

**Background:**

Syphilis in children is uncommon with the mode of infection for this rare condition likely being congenital or acquired. While most acquired cases of syphilis in children result from sexual abuse, children can also be infected with syphilis through kissing, breastfeeding, sharing of daily necessities or pre-chewed food. Here, we report a case of acquired secondary syphilis in a child due to consumption of pre-chewed-food and provide a review of the literature on the characteristics of acquired syphilis in preschool children.

**Case presentation:**

A 3-year-old girl presented with erythematous plaques and scales on her head, neck, and thighs as well as flat red papules with a moist, well circumscribed surface covered with a grayish-white film. The grandmother who cared for the girl was in the habit of pre-chewing food before giving it to the girl. The child and grandmother tested positive for RPR. The girl, who was not sexually abused, was diagnosed with acquired secondary syphilis, resulting from the transmission of pre-chewed food from her grandmother.

**Conclusions:**

Our case report and literature review reveal that close contact among family members can result in the transmission of syphilis. We recommend that pre-chewing food should be discouraged by caregivers when caring for their children to avoid disease transmission.

## Background

Syphilis is a sexually transmitted disease resulting from *Treponema pallidum* infection. The spirochete is transmitted by sexual or placental contacts [1]. Syphilis represents a disease with a long progressive duration, complex clinical manifestations, multiple organ involvements, and multiple routes and modes of transmission [2]. Children may get infected with syphilis by transplacental vaginal delivery, infected milk, or through sexual contact, in a manner similar to that of adults [3, 4]. Congenital syphilis may occur due to the lack of prenatal diagnosis in the mother, the loss of syphilitic mothers’ follow-up, the lack of medication adherence, or non-standard treatment of partners. If the infection is acquired during the lase trimester, the screening test may be negative and the newborn could get syphilis during delivery. Fetal treatment failure can also relate to treatment late in pregnancy. It may also occur in pregnant women who failed in penicillin allergy and desensitization therapy. When using macrolides, they develop drug resistance or drug components that cannot effectively pass through the placental barrier [5–9]. Syphilis can be transmitted through the fetus at any stage during pregnancy. The syphilis stage of the mother determines the possibility of vertical transmission, with transmission rates of primary and secondary syphilis being 60–100%, early latent transmission rate at 40% and a late latent transmission rate of 10% [10]. Acquired syphilis rarely occurs in early childhood, though when it does, sexual abuse is the main mode of transmission of childhood acquired syphilis [11]. However, syphilis can also be transmitted through any number of types of intimate contact, such as handling, kissing, breast-feeding and pre-chewed food [12]. Here, we describe a case of acquired childhood syphilis transmitted through non-sexual transmission and provide a review of the literature on this topic.

## Case presentation

A 3-year-old girl presented with plaques and white scales on the neck that had been present for 1 month. She was diagnosed with psoriasis at the local primary hospital and treated with topical steroid ointments, with no improvement in the skin lesions. Moreover, moist red flat patches were now observed in the perianal region during these treatments with topical ointments. As the lesions gradually increased, the caretaker brought the child to our outpatient clinic for assessment.

**Fig. 1:**
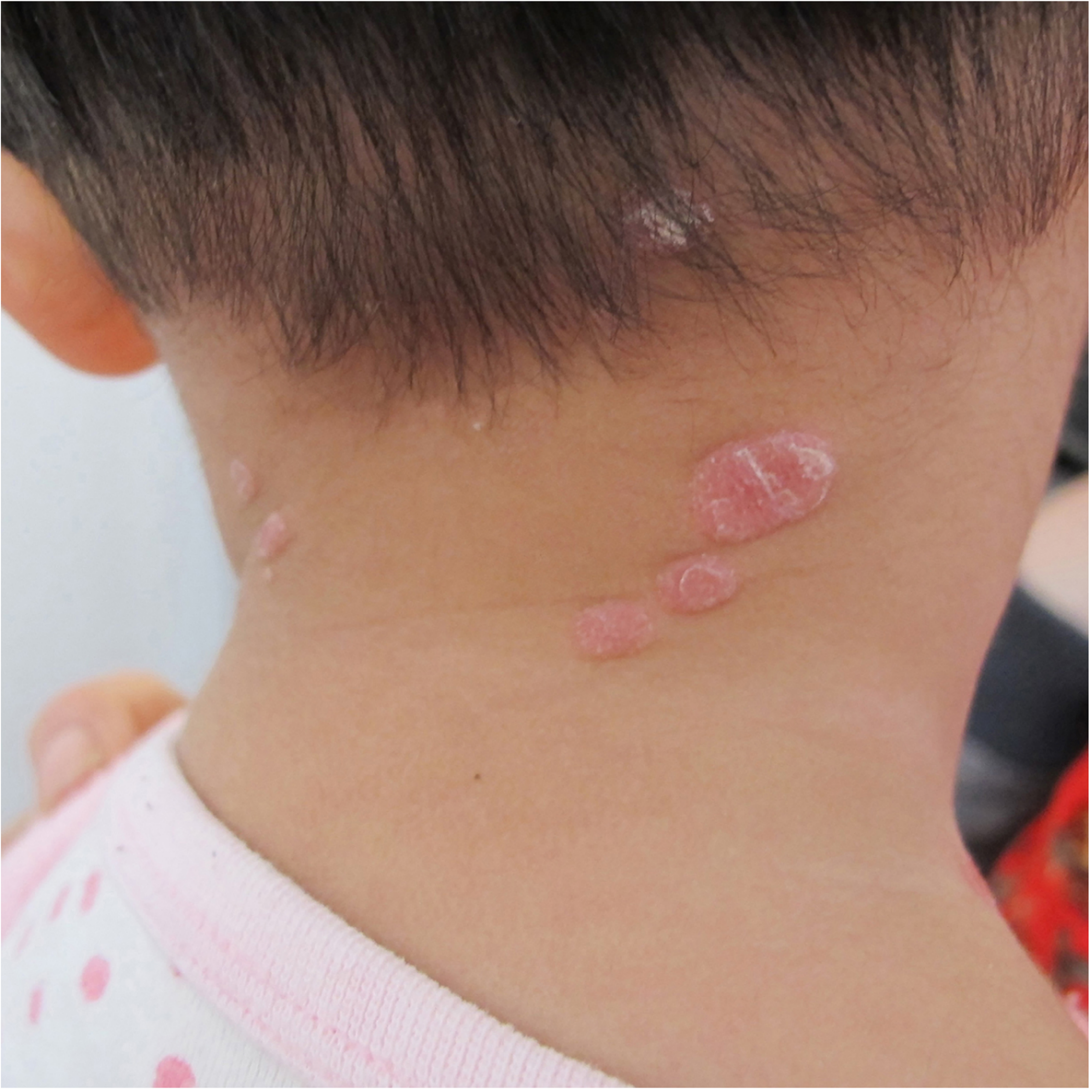
Distribution and infiltration of erythematous macules with scales on the head, neck and thighs

**Fig. 2:**
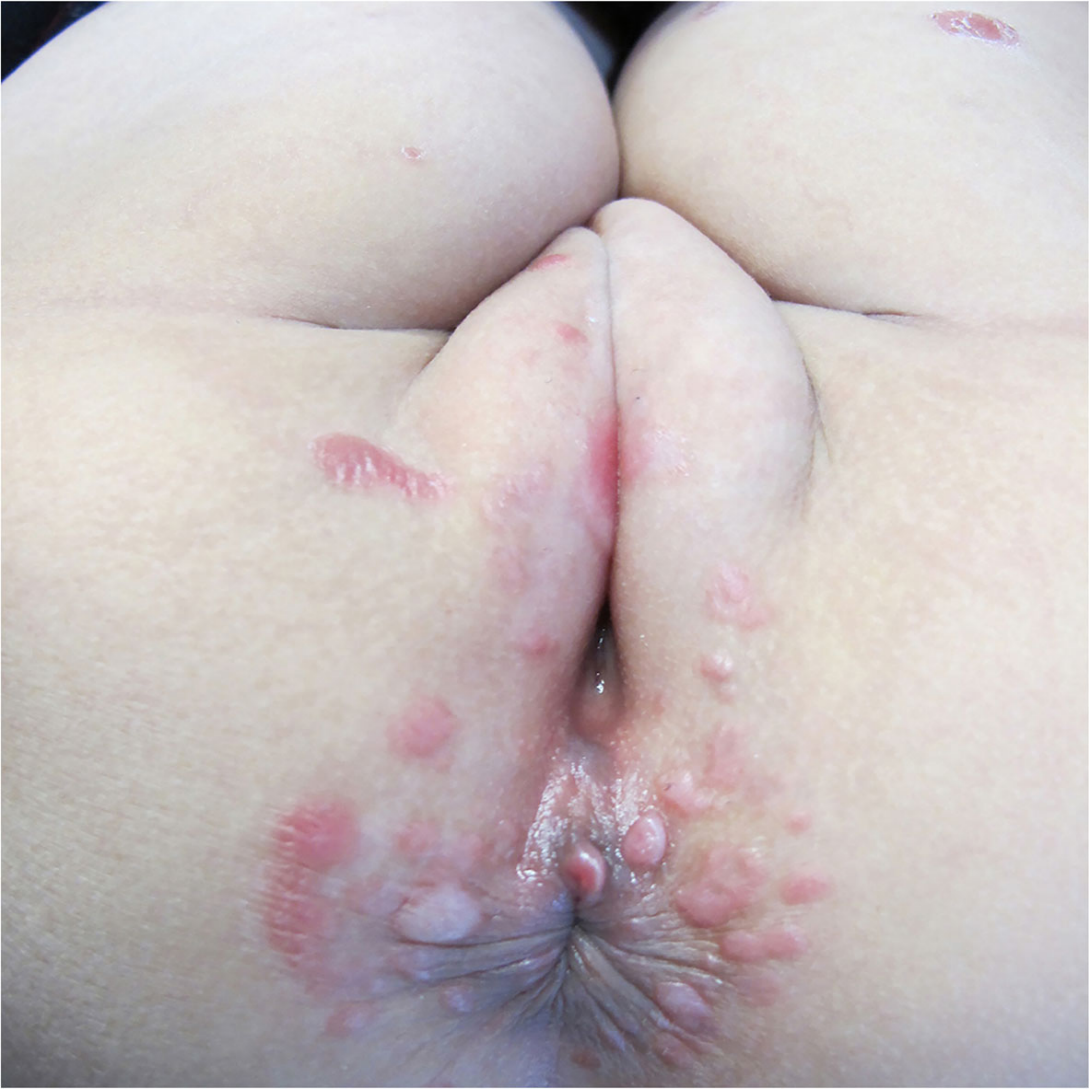
Moist and flat condylomata in vulva and perianal area

On physical examination, the child presented with erythematous plaques containing white scales on her head, neck, and thighs (Fig. 1), along with moist verrucous plaques in the vulva and perianal areas (Fig. 2). No skin lesions were observed on the palms and soles, nor was there any evidence of oral mucosal lesions, lymphadenopathy, or alopecia. Skeletal and neurological examinations were normal. While the skin lesions on the neck and thigh were suggestive of psoriasis or pityriasis rosea, the additional presence of lesions around the vulva led us to consider syphilis.

**Fig. 3:**
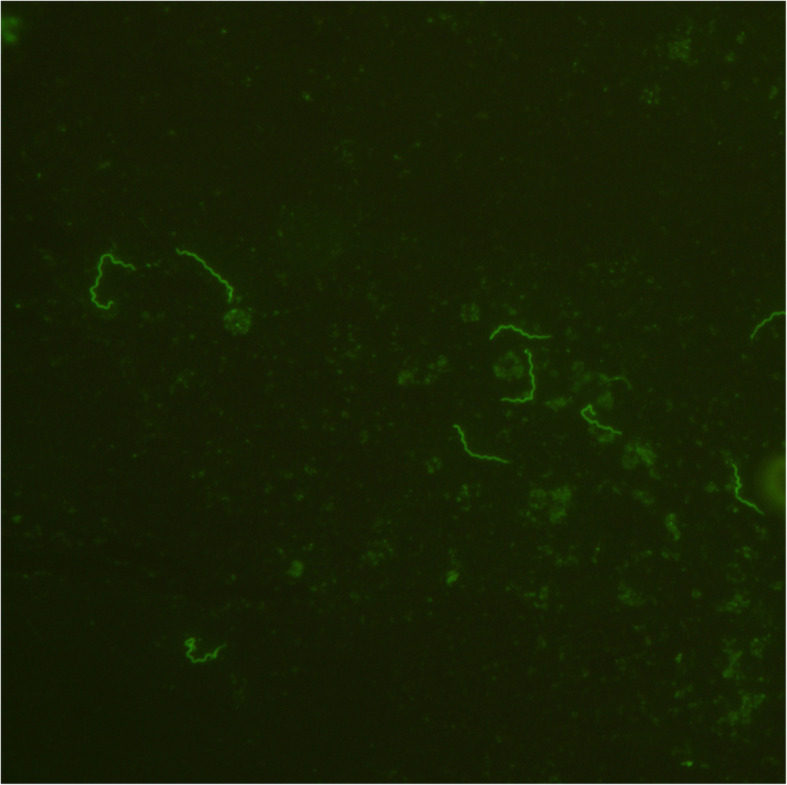
Demonstration of multiple spirochetes from a scraping smear sample of the perianal area via direct fluorescent antibody test using FITC Anti-Treponema pallidum antibody (ab20719) with the dilution of 1:10

Laboratory examination results revealed that the complete blood count, liver and renal function tests were all normal and human immunodeficiency virus (HIV) serology was negative. However, results from *T. pallidum* particle agglutination (TPPA) and the rapid plasma reagin circle test (RPR test) were positive, with the RPR test being positive at a dilution of 1:64. An examination of a scraping sample from the perineum lesions using a direct fluorescent antibody test demonstrated multiple spirochetes. This test is to label the specific anti-Treponema pallidum monoclonal antibody with FITC Anti-Treponema pallidum antibody (ab20719) at the dilution of 1:10. If *T. pallidum* is present in the specimen, it will be specifically bound by the antigen and antibody, and an apple green color can be seen under a fluorescence microscope. (Fig. 3).

The child was diagnosed as acquired secondary syphilis and treated with an intramuscular administration of benzathine penicillin at 50,000 IU/kg/week for 3 weeks according to the Chinese syphilis treatment guidelines of 2009. Three months after treatment, all lesions completely disappeared and RPR test results were now negative. Follow-up at 12 months, indicated no disease relapse and results of RPR and HIV tests were negative.

The parents of the child were astonished by the diagnosis. They reported that the child experienced a normal birth with no abnormal laboratory results at birth and they themselves denied any history of syphilis or promiscuity. Their tests for syphilis were negative. We examined the child’s hymen and anal, no signs of sexual behavior were observed. The patients and care givers also didn’t find any evidence of being sexual abused of the child in daily life. As the parents indicated that the girl was usually cared for by the grandmother, further assessments were directed to the grandmother. Results from laboratory examinations performed on the grandmother revealed that TPPA and RPR were positive, with a RPR titer of 1:64, while the physical examination failed to identify any skin lesions. When interviewing the grandmother, she indicated that she would routinely pre-chew or pre-warm the food before spoon-feeding it to the child, in this way effectively resulting in a mouth-to-mouth transmission. This feeding method continued during the period when she had acquired syphilis and been in the stage of syphilis transmission, a process which often occurs among the elderly in many countries [13].

## Discussion and conclusions

Acquired syphilis in children is, not only a medical, but social problem that has been neglected for a long time. Reviewing the literature as searched in the NCBI database over the past 30 years, searched a total of 157 articles with “child” AND “acquire syphilis” as the search strategy in NCBI, and excluding children syphilis transmitted by sexual behavior, finally revealed only eight articles with eleven cases of acquired syphilis involving non-sexual behavior (Table 1). Among these eleven cases, which included six boys and five girls, nine were from developing countries and two in developed countries. None of the cases had a history of child sexual abuse, but all cases had close contact among family members and seven cases experienced rearing under conditions involving the pre-chewing of their food. It is difficult to evaluate the incidence of children’s syphilis resulting from close family contact, therefore little attention has been directed to the study of acquired syphilis from non-sexual sources. As the painless ulcers (chancre) of primary syphilis often occur at inoculation sites [3], skin lesions in children who have acquired syphilis through consumption of pre-chewed food often appear in the oral cavity of the child. However, in sexually abused children, the chancre often occur in the perineum. Infants and young children obviously cannot convey information about their condition, as a result, the physical abnormalities present are not revealed and managed in a timely manner. Rashes associated with syphilis are often painless, nonpruritic, insensible, so it is difficult for children to actively describe physical abnormalities to caregivers, making it difficult to detect acquired syphilis by caregivers, unless the location of rashes is quite obvious. Therefore, the disease often progresses from primary to secondary syphilis in these children. An additional complication is that skin manifestations of acquired syphilis are easily confused with many other conditions, such as thrush, tonsillitis, psoriasis, cutaneous leishmaniasis and cat scratch disease [14].

**Table 1:** Clinical characteristics of cases reports of acquired syphilis in preschool children by nonsexual close contact on NCBI (1990–2021)^a^

Case, Country	Age of onset (years)	Sex	Syphilis Stage	Anatomical location (type of skin lesion)	Serological tests	potential source of infection	mechanism of transmission	Socioeconomic status and job description
1 [31] China	2	Boy	Secondary	Hair (alopecia)	RPR 1:32	Grandmother	prechewing feed and close contact	Lower classes
2 [32]. China	1.5	Boy	Secondary	Oral mucosal (moist plaque), Lymphadenopathy	RPR 1:64	Grandparent	prechewing feed and close contact	No data
3 [32]. China	10-months	Girl	Secondary	Ear (plaque)	RPR 1:4	Mother	prechewing feed and close contact	No data
4 [27]. China	2	Boy	Secondary	Hair (alopecia), perianal (condyloma lata)	RPR 1:128	Grandmother	prechewing feed and close contact	No data
5 [33]. China	2	Boy	Primary	Tongue (painless ulcer)	RPR 1:8	Grandmother	prechewing feed and close contact	No data
6 [21]. China	5	Boy	Secondary	Oral mucosal (moist plaque), trunk, palms and soles (plaque), Lymphadenopathy	RPR 1:128	Grandparents	close contact	Middle and upper classes
7 [21]. China	3	Girl	Secondary	Palm and Soles (Plaque)	RPR 1:8	Caretaker (Nanny)	close contact	Middle and upper classes
8 [21]. China	4	Girl	Secondary	Perianal (condyloma lata)	RPR 1:64	Grandparents	close contact	Middle and upper classes
9 [14]. Italy	2	Girl	Primary	Lips (painless rigid ulcer), Lymphadenopathy	RPR (+)	Mother	close contact	No data
10 [12]. America	4	Girl	Secondary	Fever, hair (alopecia), oral (plaque), palms, soles (plaque, erythematous), Lymphadenopathy	VDRL1:64	Mother	prechewing feed and close contact	Commercial sex worker
11 [34]. Germany	2	Boy	Secondary	Fever, Palms and soles (plaque), Lymphadenopathy,	TPHA (+), VDRL (+)	Mother	prechewing feed and close contact	No data

^a^We searched a total of 157 articles with “child” AND “acquire syphilis” as the search strategy in NCBI, and excluding children syphilis transmitted by sexual behavior, finally found eight case reports or case review articles

According to clinical manifestations and laboratory tests, acquired syphilis is divided into four phases, primary, secondary, latent, and tertiary syphilis. Clinical examinations for syphilis mainly include the presence of organisms as can be observed with darkfield examination of chancre exudate, serological examinations and cerebrospinal fluid assays. Serological examinations consist of nontreponemal (RPR, VDRL) and treponemal (TPPA, TPHA) tests. Nontreponemal tests, which can be used as a screening and quantitative test are characterized as having results of high sensitivity but low specificity, with the titer of RPR/VDRL being related to disease activity. Treponemal tests are highly specific and can be used for a definitive diagnosis. Treatment of syphilis should be prompt, methodological and involve use of sufficient doses [15], with the goal of achieving a clinical and serological cure. Failure to standardized treatments results in complications, the most serious of which may involve the cardiovascular and nervous systems and lead to death or serious disabilities [16]. Tertiary stage syphilis is very rare in people with normal immunity in part due to the use of antibiotics during the process of other inflammatory diseases. The treatments of choice for syphilis include penicillin G, benzyl penicillin and procaine penicillin, with dose and duration being dependent on stage and duration of the disease [3]. For children with penicillin allergies, tetracycline, doxycycline, erythromycin, azithromycin, or ceftriaxone can be used [17, 18]. Treatment failure due to the resistant to macrolides are commonly noticed.

Although most children acquire syphilis as a result of sexual abuse [19], daily close contact among family or related individuals may likely represent the main mode of nonsexual transmission of acquired syphilis in children. Nonsexual close contact such as kissing, breastfeeding, pre-chewed feeding, bathing or even the sharing of public contaminated tableware and daily necessities such as sheets, towels and shavers can all be potential sources for acquired syphilis [20, 21]. Routine interactions among infants their mothers or caregivers which can contribute to the transmission of this disease include the touching of the mother/caregiver’s mouth with their hands followed by placing the hands into their own mouths and mother/caregiver’s testing the temperature of the food with their mouth.

In addition to syphilis, administration of pre-chewed food can also serve as transmission routes for HIV [22, 23] and streptococcal pharyngitis [24]. In many countries, particularly in developing countries, the administration of pre-chewed food to infants is a custom and often occurs within older generations and individuals with low education levels. However, this practice is also present in some middle and upper class families in China [21], as it is generally considered of great importance to the child and enables increased opportunities for close contact. For infants and young children, most caretakers will start by chewing food into a puree form before feeding it to the infant. Among children infected with acquired syphilis in China, grandparents are the most likely source to infect the child via pre-chewed food. As it’s a common phenomenon for Chinese grandparents to take care of their grandchildren [25, 26]. The problem of acquired syphilis then emerges due to the 50 to 65 year olds being the most likely group to purchase commercial sex [27]. As a result, the proportion of syphilis among the elderly in China has significantly increased [28, 29]. This problem is then exacerbated by the large amount of *T. pallidum* that may spread on the surface of the oral cavity of the infected person and the relatively common incidence of periodontal disease in the elderly [30]. Even in caregivers lacking active oral lesions, pre-chewing of the food may expose infants to blood transmitted by syphilis.

Our case report and findings in the literature support the hypothesis that feeding patterns and intimate contact in daily life may represent the main mode of nonsexual transmission of syphilis in children, but the possibility of child-acquired syphilis resulting from sexual abuse must also be eliminated as an etiology. We recommend that caregivers should be to discourage prechewing of food. All caregivers should regularly inspect the child’s skin and seek prompt medical attention if any lesion is observed.

## Data Availability

The datasets used and analyzed during the current study are available from the corresponding author on reasonable request.

## Abbreviations

RPR: Rapid plasma reagin circle test
VDRL: Venereal diseases research laboratory test
TPHA: *T. pallidum* hemagglutination assay
TPPA: *T. pallidum* particle agglutination
